# Using deep learning-based natural language processing to identify reasons for statin nonuse in patients with atherosclerotic cardiovascular disease

**DOI:** 10.1038/s43856-022-00157-w

**Published:** 2022-07-15

**Authors:** Ashish Sarraju, Jean Coquet, Alban Zammit, Antonia Chan, Summer Ngo, Tina Hernandez-Boussard, Fatima Rodriguez

**Affiliations:** 1 Division of Cardiovascular Medicine and Cardiovascular Institute, Stanford University, Stanford, CA USA; 2 Department of Medicine, Stanford University, Stanford, CA USA; 3 Department of Biomedical Data Science, Stanford University, Stanford, CA USA; 4 Department of Surgery, Stanford University School of Medicine, Stanford, CA USA

**Keywords:** Cardiology, Cardiovascular diseases, Disease prevention, Health services, Computational biology and bioinformatics

## Abstract

**Background:**

Statins conclusively decrease mortality in atherosclerotic cardiovascular disease (ASCVD), the leading cause of death worldwide, and are strongly recommended by guidelines. However, real-world statin utilization and persistence are low, resulting in excess mortality. Identifying reasons for statin nonuse at scale across health systems is crucial to developing targeted interventions to improve statin use.

**Methods:**

We developed and validated deep learning-based natural language processing (NLP) approaches (Clinical Bidirectional Encoder Representations from Transformers [BERT]) to classify statin nonuse and reasons for statin nonuse using unstructured electronic health records (EHRs) from a diverse healthcare system.

**Results:**

We present data from a cohort of 56,530 ASCVD patients, among whom 21,508 (38%) lack guideline-directed statin prescriptions and statins listed as allergies in structured EHR portions. Of these 21,508 patients without prescriptions, only 3,929 (18%) have any discussion of statin use or nonuse in EHR documentation. The NLP classifiers identify statin nonuse with an area under the curve (AUC) of 0.94 (95% CI 0.93–0.96) and reasons for nonuse with a weighted-average AUC of 0.88 (95% CI 0.86–0.91) when evaluated against manual expert chart review in a held-out test set. Clinical BERT identifies key patient-level reasons (side-effects, patient preference) and clinician-level reasons (guideline-discordant practices) for statin nonuse, including differences by type of ASCVD and patient race/ethnicity.

**Conclusions:**

Our deep learning NLP classifiers can identify crucial gaps in statin nonuse and reasons for nonuse in high-risk populations to support education, clinical decision support, and potential pathways for health systems to address ASCVD treatment gaps.

## Plain language summary

Cardiovascular disease (CVD) is the leading cause of death worldwide. Statins are cholesterol-lowering drugs that are recommended by major guidelines to reduce the risk of heart attacks and death. However, despite being effective and generally well-tolerated, statins are concerningly underused, and reasons for such statin nonuse are not well-understood. Using artificial intelligence (AI) approaches that can analyze complex language data including written medical terminology, we identified statin nonuse and reasons for statin nonuse from clinical notes from electronic health records of CVD patients at a large health system. We found that nearly 2 in 5 patients lacked guideline-directed statin prescriptions. We then identified the key reasons for statin nonuse including patient (side-effects, personal preference) and clinician reasons (not practicing according to guidelines). Our AI approaches may help target interventions to address statin nonuse and thus, improve CVD treatment.

## Abstract (web summary)

Sarraju, Coquet et al. utilise a natural language processing approach to identify reasons for statin nonuse in the health records of patients with atherosclerotic cardiovascular disease (ASCVD). Their approach identifies patient-level and clinician-level reasons for statin nonuse, with differences by ASCVD type and patient race/ethnicity.

## Introduction

Atherosclerotic cardiovascular disease (ASCVD) remains the leading cause of morbidity and mortality worldwide despite the availability of numerous therapies^1^. In ASCVD, statins conclusively reduce adverse events including myocardial infarctions, strokes, and mortality^2–4^. Thus, by major guidelines including the American College of Cardiology/American Heart Association (ACC/AHA) multisociety guidelines, statins carry the strongest treatment recommendation in ASCVD in the absence of contraindications^1,5^. However, statin use in ASCVD populations is alarmingly low, with discontinuation rates approaching 50% at one year following a myocardial infarction^6,7^. Racial/ethnic minorities, women, and elderly patients are more likely to prematurely discontinue statins^8–12^. Statin nonuse is independently associated with adverse outcomes including all-cause mortality and represents an important public health gap^13^.

Reasons for statin nonuse can be multifactorial and complex, including patient, clinician, and system factors. These have typically been studied from surveys with potential selection bias and generalizability issues^14–18^. Characterizing statin nonuse directly from electronic health records (EHRs) represents a unique opportunity to capture the local epidemiology of statin nonuse in a health system, which in turn may help develop targeted interventions to improve statin use. In clinical practice, reasons for statin nonuse are documented in narrative, free-text notes in the EHR, rather than in structured fields. Identifying them requires detailed characterization of large-scale unstructured EHR data^11^.

Artificial intelligence (AI) technologies, including natural language processing (NLP), demonstrate promise in studying routine clinical data at scale, including for cardiovascular outcome prediction^19–23^. We developed a deep learning-based NLP approach (Clinical Bidirectional Encoder Representations from Transformers [Clinical BERT]) to characterize reasons for statin nonuse directly from EHR data of a multiethnic, real-world, ASCVD cohort^24^. The idea of this approach was to create a highly flexible deep learning model that can be incorporated into a clinical tool to accurately identify patients not meeting clinical guideline recommendations for statin use and then identify reasons for statin nonuse.

In this study of a multisite, multiethnic EHR-based health system, we found that approximately 40% of ASCVD patients lacked structured statin prescriptions. Using Clinical BERT, we accurately identified statin nonuse and key reasons for statin nonuse from unstructured clinical notes, including the prevalence of patient-level (side-effects, patient preference) and clinical-level (guideline-discordant practice) reasons. We observed differences in reasons for statin nonuse by patient race/ethnicity. By guiding targeted interventions to address statin nonuse, such a tool can bridge guideline-directed statin utilization gaps in diverse, real-world settings.

## Methods

### Study design

This retrospective study identified patients at Stanford Health Care Alliance (SHA) using EHR data from 1 January 2014 to 31 July 2019. SHA is an integrated health system, which includes an academic hospital (Stanford Health Care [SHC]), a community hospital (ValleyCare Hospital [ValleyCare]), and a community practice network (University Healthcare Alliance [UHA]). The study was approved by the Stanford University Institutional Review Board (Protocol 47644). Informed consent was waived under exemption 4: research on existing data.

### Patient cohort

**Fig. 1:**
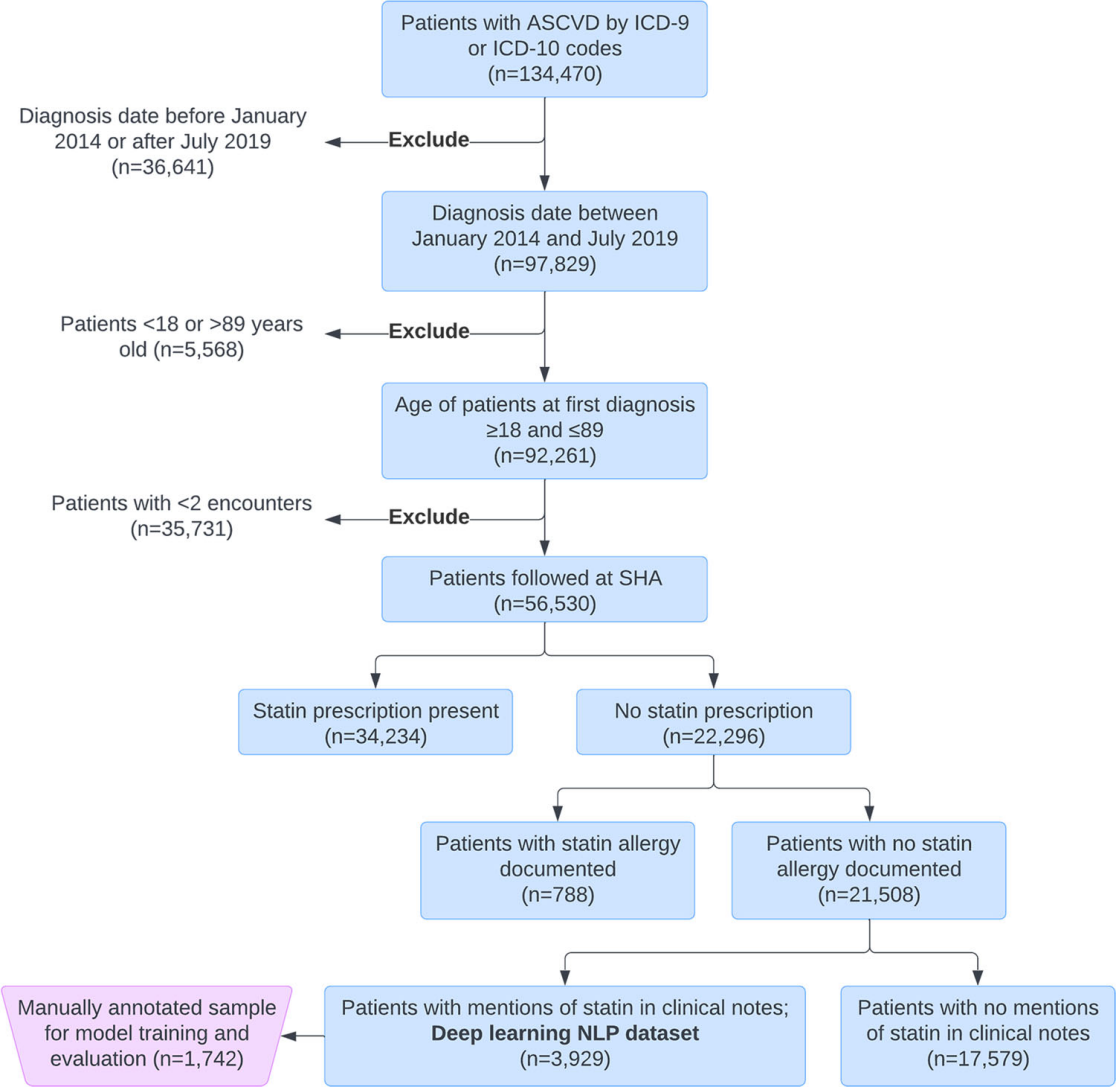
CONSORT-style diagram for cohort selection. Abbreviations: ASCVD atherosclerotic cardiovascular disease, SHA Stanford Health Care Alliance (consisting of an academic hospital, a community hospital, and a community practice clinic network), NLP natural language processing. “Statin allergy documented” refers to the documentation of a statin allergy in the structured “allergies” field of the EHR.

The study included patients diagnosed with ASCVD between the ages of 18 and 89 years between 1 January 2014 and 31 July 2019 (Fig. 1). International Classification of Diseases 9 and 10 (ICD 9 and 10) codes were used to identify the ASCVD diagnosis, which included coronary artery disease, cerebrovascular disease, peripheral arterial disease, and polyvascular disease (two or more ASCVD diagnoses; Supplementary Table 1). To ensure that patients received regular care within this healthcare system, patients were excluded if they did not have at least two encounters with an ASCVD diagnosis (Fig. 1). The first ASCVD diagnosis was considered the index diagnosis. RxNorm codes were used to identify statin medications (Supplementary Table 2). Patients with documented ASCVD diagnoses were first classified based on the presence or absence of statin prescriptions documented in structured EHR medication data at diagnosis date (Fig. 1). Statin prescriptions at diagnosis date or a new prescription within 1 month after diagnosis were included. In the case of a prescription that had no recorded end date, the prescription was considered active if it had started within 6 months prior to the index diagnosis date. Patients who lacked statin prescriptions were further classified by whether they had documented statin allergies in structured fields. Patients without documented allergies in structured fields were then further classified by the presence or absence of the term “statin” or the drug names in unstructured clinical notes (Supplementary Table 3). Types of clinical notes included were predefined and comprehensive in scope, spanning inpatient and outpatient care (Supplementary Data 1). Clinical notes dated up to 30 days after the index ASCVD diagnosis were included to allow the diagnosing providers up to 1 month after a new ASCVD diagnosis to document statin prescriptions, statin use, or statin nonuse. Encounter documentation is typically expected to be completed within 14 days in our health system. Patients who had any statin terms in clinical notes were included in the final cohort (deep learning NLP dataset, Fig. 1) for the development of the Clinical BERT models for the classification of statin nonuse and reasons for statin nonuse from unstructured clinical notes. We manually reviewed 20 random charts from patients who lacked statin terms to confirm the lack of statin use.

### Patient characteristics

Clinical data were captured at the time of index ASCVD diagnosis for patients with and without structured statin prescriptions, including age, gender, race/ethnicity, history of smoking, number of hospitalizations in the year prior to diagnosis, and comorbidities based on ICD 9 and ICD 10 codes, including heart failure, atrial fibrillation, chronic kidney disease, and liver disease. Race/ethnicity was abstracted from the EHR. ASCVD diagnoses were categorized as coronary artery disease, cerebrovascular disease, peripheral arterial disease, or polyvascular disease (Supplementary Table 1). Laboratory studies, including total cholesterol, low-density lipoprotein cholesterol (LDL-C), and creatinine kinase (CK) or creatinine phosphokinase (CPK), were obtained within 6 months of initial diagnosis, and the value closest to the diagnosis date was used. The medical specialty of the encounter visit documenting the ASCVD diagnosis was captured. The patient’s insurance payor was categorized as Medicare, Medicaid, or Private.

### Primary outcomes

The primary outcome was statin nonuse based on structured and unstructured EHR data. The secondary outcome was the reason for statin nonuse classified into the following categories: muscle-based side-effects, other side-effects, perceived lipid control, patient preference, and nonspecific reasons. The categories of “muscle-based side-effects” and “other side-effects” included documentation of side-effects attributed to statin use as well as perceived contraindications to statin use attributed to pre-existing comorbidities (examples include underlying skeletal myopathy or advanced liver disease). The category of “perceived lipid control” was defined as documentation that statin use was avoided either based on available lipid levels or deferred in favor of obtaining future lipid tests to determine the need for statin therapy despite the presence of an ASCVD diagnosis. As contemporary ASCVD guidelines recommend the initiation of statin therapy as tolerated independent of baseline lipids, this was considered a guideline-discordant practice^1^. The category of “patient preference” was defined as documentation of statin nonuse due to patient preference but without further documentation of a specific reason relevant to other categories. The category of “nonspecific” was defined as documentation of statin nonuse in clinical notes without a reason that may be included in other categories.

### Natural language processing

For the deep learning NLP dataset, as outlined above, clinical notes were obtained from a 30 day period after the index ASCVD diagnosis. For each patient, all sentences which contained mentions of statins based on a predefined statin term dictionary based on drug names (Supplementary Table 3) were concatenated in one document for model training and evaluation. We employed Clinical BERT, a novel deep learning approach consisting of semi-supervised machine learning models pretrained on a large set of free texts which can be fine-tuned to a specific target task (including text classification) in a process called transfer learning^24^. The Clinical BERT model was pretrained on notes from MIMIC III, a database containing EHRs from ICU patients at the Beth Israel Hospital in Boston, MA^24^.

### Manual annotation of reasons for statin nonuse

To create a manually annotated ground-truth dataset for NLP model training and evaluation, a sample of patients (*N* = 1742, 44%) was randomly selected from the deep learning NLP dataset. Four co-authors (AS, AC, SN, FR) manually annotated this dataset to determine whether clinical notes contained documentation of active statin use or nonuse, and subsequently extracted reasons for statin nonuse from notes according to categories. To assess reviewer concordance, the overlapping review was performed in a set of 50 patients and a set of 100 patients. Discrepancies between reviewers in these overlapping notes were resolved by repeat review by a clinician expert (AS or FR). Non-muscle side effect categories were defined more granularly prior to review (including liver side effects, gastrointestinal side effects, and neurological side effects), but were collapsed into a single category to comply with privacy regulations that prevent the reporting of groups with less than 10 participants. Categories were not considered mutually exclusive. The categories of reasons for statin nonuse were finalized upon completion of manual review.

### NLP model to classify statin nonuse

We first developed the Clinical BERT model to determine statin use versus nonuse from clinical notes in the deep learning NLP dataset; that is, among patients without statin prescriptions, we investigated clinical notes for documentation of statin use from external sources such as outside prescriptions. The fine-tuned model consisted of binary classification between patients with statin use documented in clinical notes (positive cases) and without documented statin use (negative cases).

### NLP model to classify reasons for statin nonuse

In patients without documentation of statin use in clinical notes, we developed another model to determine documented reasons for statin nonuse, classified by the categories as previously described. We fine-tuned the Clinical BERT pretrained model to perform a multiclass classification with our dataset. For this task, we implemented a two-step pipeline. First, we trained five different models to classify one class (positive cases) against the four other classes (negative cases) in a one-versus-rest strategy^25^. Second, we used a random forest model that took the probabilities of each reason from the first step as inputs to predict the final reason for nonuse. We also separately implemented a multilabel Clinical BERT classification model to predict the reason for statin nonuse.

### NLP model training and evaluation

For both NLP models to classify statin nonuse and reasons for nonuse, we split the manually annotated patients into two data sets: a training set with 80% of patients and a test set with 20% of patients. We used 10-fold cross-validation to validate the model and tune the hyperparameters (learning rate, number of epochs, ﻿strength of weight decay, and Adam’s epsilon value). To evaluate the performance of models, we assessed precision, recall, Area Under Curve (AUC) score, and F1 score. Precision (or positive predictive value) measures the fraction of correct positive predictions divided by all documents predicted as positive. Recall (or true positive rate) measures the fraction of correct positive predictions divided by the number of results that should have been predicted as positive. AUC score corresponds to the probability that a classifier will rank a positive document higher than a negative one. Finally, F1 score is defined as the harmonic mean of precision and recall. For the multiclass classifiers for the reasons for statin nonuse, we reported the weighted macro-averages of these metrics. After training and evaluation, the binary NLP model and highest performing multiclass NLP model were applied across the full deep learning NLP dataset. The distribution of reasons for statin nonuse was stratified by type of ASCVD (coronary artery disease, cerebrovascular disease, peripheral arterial disease, and polyvascular disease) and race/ethnicity (Non-Hispanic White [NHW], Non-Hispanic Black, Hispanic, and Non-Hispanic Asian).

### Statistical analysis

For comparisons of baseline characteristics between patients with and without statin prescription, we performed an unpaired *t* test for parametric data and chi-square/Fisher’s exact tests for categorical variables. Cohen’s Kappa coefficients were calculated to assess the concordance of the manual annotations between overlapping reviewers. All statistical tests were two-sided with a threshold of *p* ≤ 0.05 for statistical significance. We calculated 95% confidence intervals for NLP model performance by performing a bootstrap resampling of 1000 iterations. We also compared unadjusted and adjusted Odds Ratios (OR) of receiving high-intensity statin prescriptions (versus other statin intensities) and of receiving any statin prescriptions (versus no prescriptions) through multivariable logistic regression. Analyses were performed using Python software, version 3.7, with transformers and scikit-learn packages.

## Results

### Cohort selection

There were 56,530 patients with documented ASCVD who had at least two separate visits to the healthcare system during the study period (Fig. 1). Of these patients, 22,296 (40%) were not prescribed statins of any intensity in EHR structured medication data (Fig. 1, Table 1). Patients without a statin prescription had a mean age of 65.5 ± 14.7 years, 45.1% were women, 58.4% were NHW, 5.8% were non-Hispanic Black (Black), 9.6% were Hispanic, and 13.6% were non-Hispanic Asian (Asian). Coronary artery disease was the most common ASCVD condition among patients without a statin prescription (34.0%). Patients without a statin prescription had a baseline LDL-C level of 107.9 ± 37.2 mg/dl (mean ± standard deviation [SD]), compared with 90.2 ± 38.3 mg/dl in patients prescribed statins.

**Table 1:** Baseline characteristics of the ASCVD study cohort by the presence or absence of a statin prescription.

Characteristic at index date (*N* [% by column] unless otherwise noted; *N* = 56, 530)		Statin prescription present (*N* = 34,234)	Statin prescription absent (*N* = 22,296)	*p* value
Age (years, mean ±SD)		68.6 ± 11.6	65.5 ± 14.7	<0.001
Female		12179 (35.6%)	10054 (45.1%)	<0.001
Race	Non-Hispanic White	19086 (55.7%)	13031 (58.4%)	<0.001
	Non-Hispanic Black	1687 (4.9%)	1297 (5.8%%)	
	Hispanic	3167 (9.3%)	2139 (9.6%)	
	Non-Hispanic Asian	5553 (16.2%)	3028 (13.6%)	
	Other	3123 (9.1%)	1656 (7.4%)	
Provider location	SHC	20442 (59.7%)	11152 (50.0%)	<0.001
	UHA	12226 (35.7%)	9779 (43.9%)	
	ValleyCare	1552 (4.5%)	1191 (5.3%)	
ASCVD type	Coronary artery	20152 (58.9)	7577 (34.0%)	<0.001
	Cerebrovascular	7155 (20.9)	5484 (24.6%)	
	Peripheral Arterial	3117 (9.1)	3072 (13.8%)	
	Polyvascular	3810 (11.1%)	6163 (27.6%)	
Current smoking		2006 (5.9%)	925 (4.1%)	<0.001
Hospitalizations in prior 1 year (N)		3000 (8.8%)	1831 (8.2%)	0.022
Insurance status	Private	6984 (20.4%)	5420 (24.3%)	<0.001
	Medicare	20097 (58.7%)	11696 (52.4%)	
	Medicaid	2144 (6.3%)	1529 (6.8%)	
PCSK9 inhibitors		59 (0.2%)	68 (0.3%)	0.001
Ezetimibe		1697 (5.0%)	292 (1.3%)	<0.001
Total cholesterol (mg/dL, mean ± SD)		167.2 ± 46.2	169.3 ± 39.8	0.698
LDL-cholesterol level at index (mg/dl, mean ± SD)		90.2 ± 38.3	107.9 ± 37.2	<0.001
Chronic kidney disease		5286 (15.4%)	2248 (10.0%)	<0.001
Heart failure		6091 (17.8%)	2937 (13.2%)	<0.001
Atrial fibrillation		5565 (16.3%)	3393 (15.2%)	0.003
Liver disease		1966 (5.7%)	1483 (6.7%)	<0.001
Creatine kinase level (mean ± SD)		252.3 ± 746.6	386.5 ± 1022	0.001
Encounter specialties	Cardiology	10906 (31.8%)	7985 (35.8%)	<0.001
	Internal medicine	3176 (9.2%)	1816 (8.1%)	
	Family medicine	2263 (6.6%)	991 (4.4%)	
	Vascular surgery	1486 (4.3%)	1093 (4.9%)	
	Radiology	1181 (3.5%)	993 (4.4%)	
	Neurosurgery	664 (1.9%)	1017 (4.6%)	
	Primary care	1106 (3.2%)	459 (2.0%)	
	Emergency medicine	977 (2.8%)	540 (2.4%)	
	Neurology	802 (2.3%)	618 (2.8%)	
	Anesthesiology	791 (2.3%)	510 (2.3%)	
	Others	10882 (31.8%)	6274 (28.1%)	

*ASCVD* atherosclerotic cardiovascular disease, *LDL* low-density lipoprotein, *SHC* Stanford Health Care (academic hospital), *UHA* University Health Alliance (community practice network), *ValleyCare* ValleyCare Hospital (community hospital), *SD* standard deviation.

Of 22,296 patients without statin prescriptions, a total of 788 patients (3.5%) had documented statin allergies in the structured portion of the EHR. A manual review of 20 random patients with structured allergies confirmed the presence of allergies and lack of active statin use. Among the remaining 21,508 patients, a total of 17,579 (81.7%) had no mention of statin terms in clinical notes. The remaining 3929 patients who had any mention of statin terms in their clinical notes formed the deep learning NLP dataset for Clinical BERT model development and evaluation (Fig. 1).

### Statin prescriptions

Across all 56,530 patients with ASCVD, women (compared with men), community hospital patients (compared with an academic hospital), and patients with cerebrovascular disease, peripheral arterial disease, or polyvascular disease (compared with coronary artery disease) were less likely to receive any statin prescriptions (Supplementary Table 4). Women (compared with men) and those with cerebrovascular disease or peripheral arterial disease (compared with coronary artery disease) were less likely to receive high intensity statin prescriptions (Supplementary Table 5).

### Manual annotation results

**Fig. 2:**
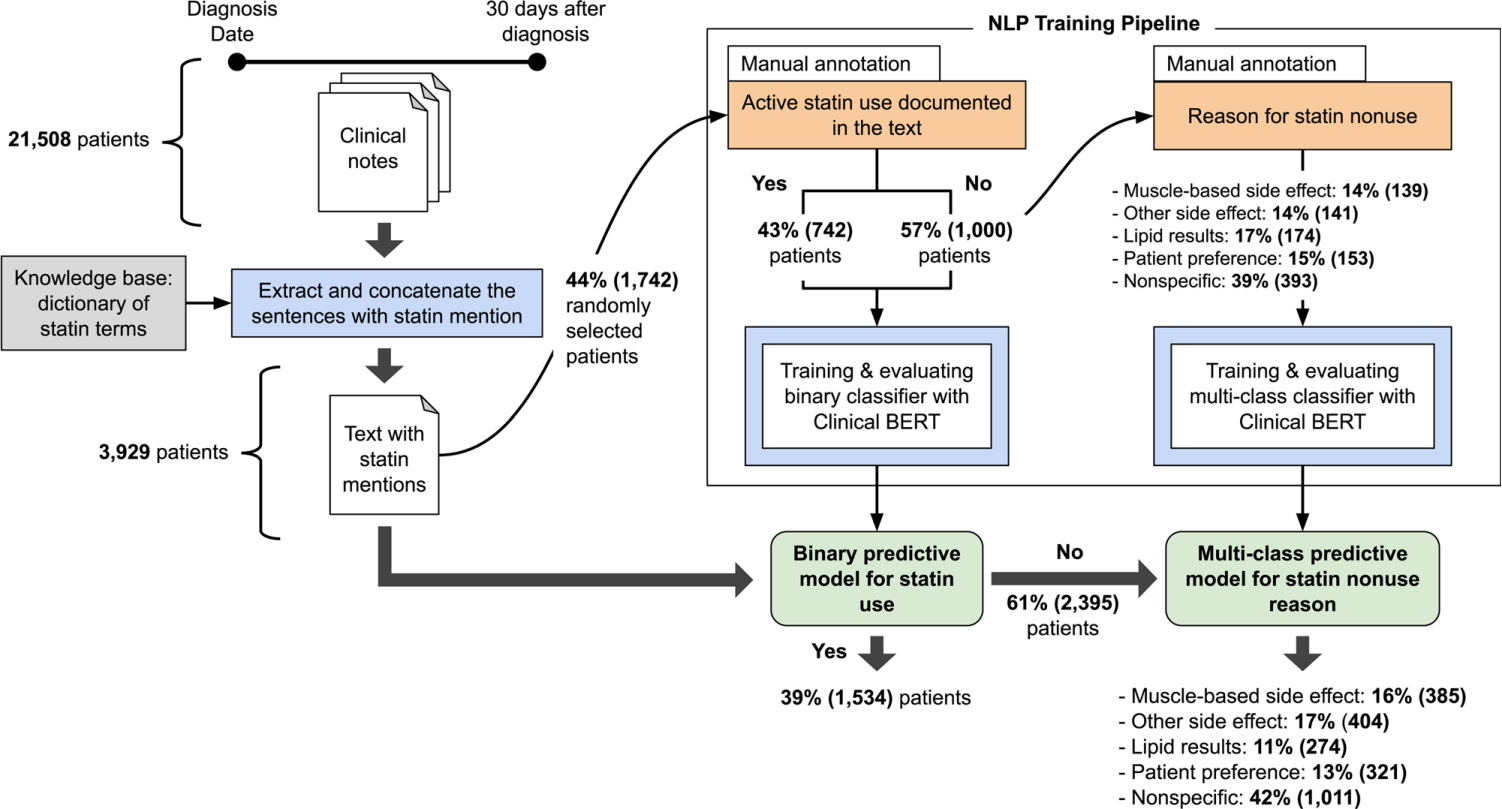
Training, internal validation, and application of a deep learning model (Clinical BERT) for natural language processing to identify statin nonuse and classify reasons for statin nonuse from unstructured clinical notes of patients with ASCVD. Abbreviations: BERT Bidirectional Encoder Representations from Transformers, NLP natural language processing.

In the manually annotated dataset (Fig. 2), 742 (43%) were on statins per unstructured notes despite no prescription data. Among the 1000 (57%) statin nonusers, reasons for nonuse included perceived lipid control (17%, *N* = 174), muscle side-effects (14%, *N* = 139), and patient preference (15%, *N* = 153) (Fig. 2). Kappa coefficients for overlapping manual review of 50-patient and 100-patient subsets were 0.91 and 0.99, respectively, indicating strong reviewer agreement.

### NLP model evaluation

In the held-out test set of the manually annotated dataset, the binary NLP model classified statin nonuse with an overall AUC of 0.94 (95% CI 0.93–0.96) (Table 2). Among statin nonusers, the two-step model classified reasons for statin nonuse with an overall weighted-average AUC of 0.88 (95% CI 0.86–0.91). The simple multilabel classification model classified reasons for statin nonuse with an overall AUC of 0.86 (95% CI 0.82–0.89).

**Table 2:** Performance of deep learning NLP models to characterize statin nonuse from unstructured clinical notes in persons with ASCVD.

Task	Dataset	Precision*	Recall*	F1 score*	AUC*
Binary classification of statin use	10-fold cross-validation (N = 1,393)	0.88 (0.86–0.90)	0.82 (0.77-0.87)	0.85 (0.83–0.87)	0.94 (0.93–0.95)
	Test set (N = 349)	0.87 (0.82–0.91)	0.82 (0.76–0.88)	0.84 (0.81–0.88)	0.94 (0.93–0.96)
Two-step classifier* for statin nonuse reasons	10-fold cross-validation (N = 800)	0.63 (0.59–0.65)	0.62 (0.54–0.72)	0.62 (0.59–0.64)	0.84 (0.81–0.85)
	Test set (N = 200)	0.68 (0.63–0.75)	0.69 (0.60–0.79)	0.68 (0.62–0.75)	0.88 (0.86–0.91)
Multilabel classification of statin nonuse reasons (simple mutlilabel model)	10-fold cross-validation (N = 800)	0.60 (0.58–0.64)	0.61 (0.56–0.66)	0.59 (0.56–0.63)	0.85 (0.83–0.87)
	Test set (N = 200)	0.64 (0.61–0.70)	0.66 (0.60–0.73)	0.64 (0.58–0.71)	0.86 (0.82–0.89)

^*^The two-step classifier represents the predicted probabilities of multiple classifiers (each reason for statin nonuse versus others) reconciled by a Random Forest.

*ASCVD* atherosclerotic cardiovascular disease, *NLP* natural language processing.

### NLP model application in the full cohort and by pre-specified subgroups

After NLP model development and evaluation, the binary statin nonuse model and the hurdle multiclass model for reasons for statin nonuse were applied to the full dataset of 3929 patients (Fig. 2). The models found that 1534 (39%) of these patients were statin users based on their clinical notes despite no documented statin prescriptions. Among the remaining 2395 statin nonusers, reasons for statin nonuse included muscle-based side-effects (16%), perceived lipid control (11%), and patient preference (13%; Fig. 2).

Reasons for statin nonuse were further stratified by type of ASCVD and by race/ethnicity (Table 3). Non-Hispanic black, Non-Hispanic Asian, and Hispanic patients had higher representation in the perceived lipid control group (a guideline-discordant practice) when compared with the other groups (*p* < 0.001). Patients with cerebrovascular disease had a higher representation in the perceived lipid control group when compared with other groups (*p* < 0.001). Excerpts denoting each reason for statin nonuse are outlined in Table 4.

**Table 3:** NLP-identified reasons for statin nonuse in patients with ASCVD, stratified by type of ASCVD and race/ethnicity.

Cohort (*N* = 3929)	Reason for nonuse					*p* value
	Side effect					
	Muscle	Other	Nonspecific	Perceived lipid control	Patient preference	
Total number	385	404	1011	274	321	
Stratified by type of ASCVD (*N*, % by column)						
Coronary artery disease	224 (58.2)	233 (57.7)	472 (46.7)	123 (44.9)	175 (54.5)	<0.001
Peripheral artery disease	35 (9.1)	38 (9.4)	129 (12.8)	29 (10.6)	47 (14.6)	
Cerebrovascular disease	87 (22.6)	85 (21.0)	310 (30.7)	95 (34.7)	74 (23.1)	
Polyvascular disease	39 (10.1)	48 (11.9)	100 (9.9)	29 (10.6)	47 (14.6)	
Stratified by race/ethnicity (*N*, % by column)						
Non-Hispanic White	244 (63.4)	273 (67.6)	554 (54.8)	132 (48.2)	214 (66.7)	<0.001
Non-Hispanic Black	13 (3.4)	25 (6.2)	62 (6.1)	19 (6.9)	13 (4.0)	
Hispanic	30 (7.8)	15 (3.7)	96 (9.5)	34 (12.4)	19 (5.9)	
Non-Hispanic Asian	40 (10.4)	46 (11.4)	96 (9.5)	34 (12.4)	19 (5.9)	

*ASCVD* atherosclerotic cardiovascular disease, *NLP* natural language processing.

**Table 4:** Excerpts from clinical notes demonstrating the reasons for statin nonuse identified in this study.

Category	Note excerpt
Muscle-based side-effects	“intolerant of low dose statins (started with high CK)”
Other side-effects	“has been intolerant to 3 different statin drugs... they cause diarrhea and nausea”
Perceived lipid control	“Will also check lipid panel. If LDL < 100, no indication for statin at this time”; “… LDL is well controlled”
Patient Preference	“Declines statins”
Nonspecific	“OK for no statin at this time”; “discuss statin next visit”

*ASCVD* atherosclerotic cardiovascular disease, *CK* creatinine kinase, *LDL* low-density lipoprotein cholesterol, *NLP* natural language processing.

Among patients without structured statin prescriptions, individuals who were statin users according to NLP were slightly more likely to be male and have coronary artery disease compared with statin nonusers according to NLP (Supplementary Table 6).

## Discussion

Strong evidence conclusively supports the use of statins in patients with ASCVD to reduce cardiovascular morbidity and mortality. However, in a real-world, multiethnic cohort of ASCVD patients, approximately 40% lacked guideline-concordant statin prescriptions. There was limited clinical documentation of statin nonuse overall. A deep learning NLP approach (Clinical BERT) reliably classified statin nonuse and reasons for statin nonuse from large-scale unstructured notes including patient-level (side-effects, patient preference) and clinical-level reasons (guideline-discordant practice).

The benefits of statin use in ASCVD are well-established^1^; yet, there remain major gaps in statin utilization^6,7^. Our study adds to this literature by highlighting a high proportion of ASCVD patients without documented statin prescriptions in a large health system, including disparities in statin prescriptions by gender, race/ethnicity, and practice setting (academic versus community-based). There is a strong need to develop interventions to bridge statin utilization gaps. Previous work studying reasons for statin nonuse has often relied on surveys that are potentially limited by selection bias, generalizability, and scalability^15,16^. A prior EHR-based study addressed statin discontinuation among patients with existing prescriptions and used a rules-based approach to analyze notes^11^. To our knowledge, our study is the first to employ a deep learning-based NLP approach to comprehensively track statin nonuse and reasons for statin nonuse across structured and unstructured EHR data. We capture statin nonuse in a multiethnic multisite health system in detail, including documentation gaps, clinician- and patient-level reasons for nonuse, and differences by characteristics such as ASCVD type and race/ethnicity.

EHR-based studies of medication utilization often rely on structured data such as prescriptions. However, we found substantial discordance between structured and unstructured EHR documentation of statin use. Inconsistent documentation of external prescriptions and medical care may contribute to this finding^26^ For example, we found clinical free-text documentation of active statin use in patients without statin prescriptions, suggesting that statin prescriptions were an unreliable surrogate for statin use in our cohort. Statin allergies in structured data were infrequently documented, but limiting side-effects were documented for patients without structured statin allergies, suggesting that using structured allergy data to infer statin intolerance would have been inaccurate. These results highlight the need for a standardized approach to document medication use in EHRs, a recognized issue for real-world data^27^. Importantly, research efforts that study real-world medication utilization may need to mitigate the limitations of structured data by incorporating unstructured data at scale, as in our study.

Our study highlights poor clinical documentation of statin nonuse, with only 18% of patients without statin prescriptions demonstrating any mention of statins in clinical notes. These findings suggest important opportunities to improve statin nonuse documentation, for example, through smart text phrases or similarly integrated user-friendly tools that are guided by NLP. Ensuring adequate documentation is mandatory in learning health systems for quality improvement (QI) and research efforts that rely on large-scale records. A deep learning NLP pipeline like ours may be well-suited to study and bridge documentation gaps by leveraging unstructured EHR data^28^.

Accurately characterizing reasons for nonuse is critical to closing statin utilization gaps. An approach like ours may enhance efforts to improve statin utilization by (1) identifying reasons for nonuse to guide targeted clinical decision support tools, (2) identifying disparities by relevant clinical factors such as type of ASCVD, and (3) providing a pathway for EHR-driven statin utilization QI or research efforts. For example, patients with documented muscle-based side-effects could be flagged for further study using validated tools to confirm statin-related symptoms and to develop targeted interventions to re-challenge statins or address nocebo effects^17,29^. Differences in statin nonuse by factors such as race/ethnicity or type of ASCVD may help guide equitable statin implementation strategies^30^. Future work should prospectively explore the role of NLP-guided interventions to promote statin utilization.

Overall, the goal of our study was to build an accurate AI-based model to classify statin nonuse and reasons for nonuse among patients diagnosed with ASCVD. Using data derived from real-time EHRs, we trained our models to identify a patient that does not have any documentation of statin use for their ASCVD, and to identify reasons for statin nonuse. Such models are well-positioned to help overcome the challenges of real-world statin utilization by providing a better understanding of statin nonuse at scale. Such information will be a step forward towards improved statin use in routine care that is urgently needed to help reduce ASCVD disparities and gaps across diverse populations.

The study should be interpreted in the context of its limitations. Our cohort was a population from Northern California that may not reflect other ASCVD populations across the United States. However, our cohort is comprised of diverse practice sites including an academic hospital (SHC), a community hospital (ValleyCare), and a community practice network (UHA). We grouped non-muscle-based side-effects into a single category for the NLP model due to their low frequency and because we were unable to report outcomes with 10 or fewer participants due to HIPAA privacy regulations. It is possible that patients received additional care from outside health systems, which is a limitation of a single health system analysis. However, to mitigate the effects of care fragmentation and ensure regular care in our health system, we included patients with a new ASCVD diagnosis recorded in our system and at least 2 clinic visits associated with that diagnosis. Our EHR medication reconciliation section also includes medications prescribed outside of our health system and medications that are self-reported by patients. Future work should consider characterizing the frequency of more discrete categories of side-effects. We were unable to disaggregate racial/ethnic groups further or include socioeconomic information due to data limitations. Due to low overall documentation, the sample sizes of stratified groups (for example, muscle side-effects stratified by type of ASCVD) were small, and these results should therefore be interpreted with caution and considered hypothesis-generating. While Clinical BERT was pretrained on external notes and we report model performance from a held-out test set, external validation, as well as a prospective study of NLP-guided interventions, is needed to ensure generalizability, outcome benefit, and cost-effectiveness prior to wide deployment.

In conclusion, in multiethnic ASCVD patients in a multisite health system, we observed suboptimal statin prescription rates and limited clinical documentation of statin nonuse. A deep learning NLP approach (Clinical BERT) reliably identified statin nonuse and reasons for nonuse from unstructured EHRs, including patient-level factors (side-effects, patient preference) and clinician-level factors (guideline-discordant practice). Advanced NLP approaches may help to learn health systems to leverage clinical documentation and characterize reasons for statin nonuse at scale from EHRs, thus potentially providing a pathway to address important ASCVD treatment gaps.

### Reporting summary

Further information on research design is available in the Nature Research Reporting Summary linked to this article.

## ^Supplementary information^

Supplementary Information

Reporting Summary

Supplementary Data 1

Description of Additional Supplementary Files

## Data Availability

The datasets analyzed during the current study are not publicly available due to reasonable privacy and security concerns. The underlying EHR data are not easily redistributable to researchers other than those engaged in the Institutional Review Board-approved research collaborations in the current project. The corresponding author may be contacted for access to EHR data for an IRB-approved collaboration.

## Code availability

The code can be accessed at 10.25740/gn923yy3398^31^.
