# Supplementary material for: Using deep learning-based natural language processing to identify reasons for statin nonuse in patients with atherosclerotic cardiovascular disease
Source: Commun Med (Lond). 2022 Jul 15;2:88. doi: 10.1038/s43856-022-00157-w (PMC9287295; doi:10.1038/s43856-022-00157-w)
Supplement: Supplementary file 1 — Supplementary Information [file 43856_2022_157_MOESM1_ESM.pdf]

SUPPLEMENTARY INFORMATION

Using Deep Learning-based Natural Language Processing to Identify Reasons for Statin Nonuse in Patients with Atherosclerotic Cardiovascular Disease

Ashish Sarraju[1*], Jean Coquet [2,3*], Alban Zammit [2,3], Antonia Chan [2], Summer Ngo [1], Tina Hernandez-Boussard [2,3,4**], Fatima Rodriguez[1**]

[1]Division of Cardiovascular Medicine and Cardiovascular Institute, Stanford University, Stanford, CA, USA
[2]Department of Medicine, Stanford University, Stanford, CA, USA
[3]Department of Biomedical Data Science, Stanford University, Stanford, CA, USA
[4]Department of Surgery, Stanford University School of Medicine, Stanford, CA, USA

* These authors contributed equally

** These authors jointly supervised this work

Supplementary Table 1. ICD codes of ASCVD diagnoses.

**Condition**	**ICD 9 codes**	**ICD 10 codes**
Coronary Artery Disease	410.*, 411.*, 412.*, 413.*, 414.*	I22.8, I22.1, I21.4, I21.11, I22.2, I21.A1, I21.9, I23.3, I22.0, I21.A9, I21.3, I21.02, I21.01, I21.21, I22.9, I21.29, I21.19, Z95.5, I20.*, I23.7, I24.*, I25.*, T82.85, I70.*, Z98.61
Cerebrovascular Disease	433.*,434.*, 435.*, 436.*, 437.*, 438.*	I63.031, I65.09, I63.00, I63.02, I63.032, I65.01, I65.01, I63.012, I63.09, I63.039, I65.02, I63.033, I65.03, I63.011, I63.019, I63.013, I66.03, I66.09, I67.89, I66.01, I66.02, I67.858, I67.2, I67.82, I67.81, G45.*, G46.*, I63.*, I67.85, I67.8, I67.9, I69.30, I65.*, I66.*, I68.*, I68.8
Peripheral vascular disease	440.*	I70.*, I73.9, I73.8, I74.4, I75.011, I75.012, I75.013, I75.019, I75.021, I75.022, I75.023, I75.029, I75.89

Supplementary Table 2. RxNorm codes for statin medications

Medication name	RxCUI codes
Lovastatin	6472
Pitavastatin	861634
Fluvastatin	41127
Rosuvastatin	301542
Pravastatin	42463
Atorvastatin	83367
Simvastatin	36567

Supplementary Table 3. Statin term dictionary.

Statin
Atorvastatin
Fluvastatin
Lovastatin
Pitavastatin
Pravastatin
Rosuvastatin
Simvastatin
Altocor
Altoprev
Crestor
Juvisync
Lescol
Lipitor
Livalo
Mevacor
Pravachol
Zocor

Supplementary Table 4. Predictors of statin use (versus no statin use) among patients with ASCVD diagnoses.

Variables		Unadjusted		Adjusted	
		OR (95% CI)	p	OR (95% CI)	p
**Age*** (N = 56,530)		1.19 (1.18 - 1.21)	<0.001	1.28 (1.26 - 1.31)	<0.001
**Gender** (N = 34,291; Ref: Male)	Female (N = 22,233)	0.67 (0.65 - 0.70)	<0.001	0.67 (0.64 - 0.70)	<0.001
**Race/Ethnicity** (N = 32,117; Ref: Non-Hispanic White)	Non-Hispanic Black (N = 2,984)	0.89 (0.82 - 0.96)	0.002	1.10 (1.01 - 1.21)	0.033
	Hispanic (N = 5,306)	1.01 (0.95 - 1.07)	0.72	1.12 (1.05 - 1.21)	0.001
	Non-Hispanic Asian (N = 8,581)	1.25 (1.19 - 1.32)	<0.001	1.22 (1.15 - 1.29)	<0.001
	Other (N = 4,779)	1.29 (1.21 - 1.37)	<0.001	1.20 (1.11 - 1.29)	<0.001
**Provider Location** (N = 31,594; Ref: SHC)	UHA (N = 22,005)	0.68 (0.66 - 0.71)	<0.001	0.70 (0.67 - 0.73)	<0.001
	ValleyCare (N = 2,743)	0.71 (0.66 - 0.77)	<0.001	0.70 (0.64 - 0.77)	<0.001
**Type of ASCVD** (N = 27,729; Ref: Coronary Artery Disease**)**	Cerebrovascular (N = 12,639)	0.49 (0.47 - 0.51)	<0.001	0.54 (0.51 - 0.57)	<0.001
	Peripheral Arterial (N = 6,189)	0.38 (0.36 - 0.40)	<0.001	0.38 (0.35 - 0.40)	<0.001
	Polyvascular (N = 9,973)	0.23 (0.22 - 0.24)	<0.001	0.24 (0.23 - 0.26)	<0.001
**Insurance status** (N = 31,793, Ref: Medicare)	Medicaid (N = 3,673)	0.82 (0.76 - 0.87)	<0.001	1.00 (0.93 - 1.09)	0.87
	Private (N = 12,404)	0.75 (0.72 - 0.78)	<0.001	0.99 (0.94 - 1.05)	0.735

Supplementary Table 5. Predictors of high-intensity statin therapy (versus low or moderate intensity therapy) in patients with ASCVD diagnoses.

Variables		Unadjusted		Adjusted	
		OR (95% CI)	p	OR (95% CI)	p
**Age***<br>**(N = 33,371)**		0.79 (0.78 - 0.81)	<0.001	0.84 (0.82 - 0.87)	<0.001
**Gender (N = 21,518; Ref: Male)**	Female (N = 11,852)	0.72 (0.68 - 0.75)	<0.001	0.77 (0.73 - 0.82)	<0.001
**Race/Ethnicity (N = 18,575; Ref: Non-Hispanic White)**	Non-Hispanic Black (N = 1,655)	1.29 (1.17 - 1.43)	<0.001	1.27 (1.13 - 1.43)	<0.001
	Hispanic (N = 3,094)	1.15 (1.07 - 1.24)	<0.001	1.02 (0.94 - 1.12)	0.614
	Non-Hispanic Asian (N = 5,431)	0.88 (0.83 - 0.94)	<0.001	0.82 (0.76 - 0.88)	<0.001
	Other (N = 3,032)	1.22 (1.13 - 1.32)	<0.001	1.07 (0.98 - 1.17)	0.118
**Provider Location (N = 19,765; Ref: SHC)**	UHA<br>(N = 12,055)	0.94 (0.90 - 0.99)	0.009	0.96 (0.91 - 1.01)	0.156
	ValleyCare<br>(N = 1,537)	1.05 (0.95 - 1.17)	0.352	1.07 (0.95 - 1.20)	0.267
**Type of ASCVD (N = 19,709; Ref: Coronary Artery Disease)**	Cerebrovascular (N = 6,919)	0.82 (0.77 - 0.86)	<0.001	0.87 (0.81 - 0.92)	<0.001
	Peripheral Arterial (N = 3,017)	0.47 (0.43 - 0.51)	<0.001	0.53 (0.48 - 0.58)	<0.001
	Polyvascular (N = 3,726)	1.25 (1.17 - 1.34)	<0.001	1.34 (1.23 - 1.45)	<0.001
**Insurance status (N = 19,552; Ref: Medicare)**	Medicaid (N = 2,113)	1.97 (1.80 - 2.16)	<0.001	1.63 (1.48 - 1.81)	<0.001
	Private (N = 6,817)	1.61 (1.52 - 1.70)	<0.001	1.19 (1.11 - 1.27)	<0.001

Supplementary Table 6. Characteristics of patients without structured statin prescriptions, stratified by NLP-identified use or nonuse of statins documented in clinical notes.

		Not using statins per NLP	Using statins per NLP	P-Value
Number of patients, n (%)		20762 (93.1)	1534 (6.8)	
Age, mean (SD)		65.4 (14.7)	68.1 (12.0)	<0.001
Gender, n (%)	Female	9416 (45.4)	569 (37.1)	<0.001
	Male	11341 (54.6)	965 (62.9)	
Race/Ethnicity, n (%)	Hispanic/Latino	1997 (9.6)	127 (8.3)	0.011
	Missing	1074 (5.2)	75 (4.9)	
	Non-Hispanic Asian	2834 (13.6)	229 (14.9)	
	Non-Hispanic Black	1219 (5.9)	63 (4.1)	
	Non-Hispanic White	12083 (58.2)	934 (60.9)	
	Other	1555 (7.5)	106 (6.9)	
Provider Location, n (%)	SHC	10530 (50.7)	837 (54.6)	<0.001
	UHA	8987 (43.3)	614 (40.0)	
	Valleycare	1077 (5.2)	83 (5.4)	
ASCVD type, n (%)	Cerebrovascular	5130 (24.7)	306 (19.9)	<0.001
	Coronary artery	7193 (34.6)	688 (44.9)	
	Peripheral Arterial	2896 (13.9)	118 (7.7)	
	Polyvascular	5543 (26.7)	422 (27.5)	
Current Smoking, n (%)		867 (4.2)	71 (4.6)	0.432
Hospitalizations in prior 1 year, n (%)		1758 (8.5)	156 (10.2)	0.025
Insurance status, n (%)	Medicaid	1438 (6.9)	77 (5.0)	<0.001
	Medicare	10870 (52.4)	918 (59.8)	
	Other/missing	3397 (16.4)	216 (14.1)	
	Private	5057 (24.4)	323 (21.1)	
Ezetimibe, n (%)		306 (1.5)	226 (14.7)	<0.001
Chronic Kidney Disease, n (%)		2109 (10.2)	212 (13.8)	<0.001
Heart Failure, n (%)		2780 (13.4)	244 (15.9)	0.013
Atrial fibrillation, n (%)		3175 (15.3)	283 (18.4)	0.004
Liver disease, n (%)		1415 (6.8)	102 (6.6)	0.844
