# Supplementary material for: Using deep learning-based natural language processing to identify reasons for statin nonuse in patients with atherosclerotic cardiovascular disease
Source: Commun Med (Lond). 2022 Jul 15;2:88. doi: 10.1038/s43856-022-00157-w (PMC9287295; doi:10.1038/s43856-022-00157-w)
Supplement: Supplementary file 2 — Reporting Summary [file 43856_2022_157_MOESM2_ESM.pdf]

nature portfolio

Corresponding author(s): Fatima Rodriguez

Last updated by author(s): Jun 27, 2022

Reporting Summary

Nature Portfolio wishes to improve the reproducibility of the work that we publish. This form provides structure for consistency and transparency in reporting. For further information on Nature Portfolio policies, see our Editorial Policies and the Editorial Policy Checklist.

Statistics

For all statistical analyses, confirm that the following items are present in the figure legend, table legend, main text, or Methods section.

n/a	Confirmed	
☐	☒	The exact sample size (*n*) for each experimental group/condition, given as a discrete number and unit of measurement
☐	☒	A statement on whether measurements were taken from distinct samples or whether the same sample was measured repeatedly
☐	☒	The statistical test(s) used AND whether they are one- or two-sided<br>*Only common tests should be described solely by name; describe more complex techniques in the Methods section.*
☒	☐	A description of all covariates tested
☒	☐	A description of any assumptions or corrections, such as tests of normality and adjustment for multiple comparisons
☐	☒	A full description of the statistical parameters including central tendency (e.g. means) or other basic estimates (e.g. regression coefficient) AND variation (e.g. standard deviation) or associated estimates of uncertainty (e.g. confidence intervals)
☐	☒	For null hypothesis testing, the test statistic (e.g. *F*, *t*, *r*) with confidence intervals, effect sizes, degrees of freedom and *P* value noted<br>*Give P values as exact values whenever suitable.*
☒	☐	For Bayesian analysis, information on the choice of priors and Markov chain Monte Carlo settings
☒	☐	For hierarchical and complex designs, identification of the appropriate level for tests and full reporting of outcomes
☐	☒	Estimates of effect sizes (e.g. Cohen's *d*, Pearson's *r*), indicating how they were calculated

Our web collection on statistics for biologists contains articles on many of the points above.

Software and code

Policy information about availability of computer code

Data collection	We used Oracle Software SQL Developer to execute the SQL code and extract the data from a copy of CLARITY database, which is an application of EPIC (electronic medical record software) that stores the patient data in a relational schema.
Data analysis	Data analysis was performed with Python software, version 3.7, with transformers and scikit-learn packages.

For manuscripts utilizing custom algorithms or software that are central to the research but not yet described in published literature, software must be made available to editors and reviewers. We strongly encourage code deposition in a community repository (e.g. GitHub). See the Nature Portfolio guidelines for submitting code & software for further information.

Data

Policy information about availability of data

All manuscripts must include a data availability statement. This statement should provide the following information, where applicable:

- Accession codes, unique identifiers, or web links for publicly available datasets
- A description of any restrictions on data availability
- For clinical datasets or third party data, please ensure that the statement adheres to our policy

The datasets analyzed during the current study are not publicly available: due to reasonable privacy and security concerns, the underlying EHR data are not easily redistributable to researchers other than those engaged in the Institutional Review Board-approved research collaborations in the current project. The corresponding author may be contacted for access to EHR data for an IRB approved collaboration.

Human research participants

Policy information about studies involving human research participants and Sex and Gender in Research.

Reporting on sex and gender	No exclusions were made based on sex/gender. Of 56,530 patients included in our analysis, 22,233 (39.3%) were female based on self-reported electronic health record data. From this cohort, 34,234 patients had a statin prescription present and 12,179 (35.6%) were female. There were 22,296 patients without a statin prescription and 10,054 (45.1%) were female.
Population characteristics	All patients included in the study cohort had an ASCVD diagnosis. Of patients with a statin prescription, the average age was 68.6 ± 11.6 years, and 55.7% were non-Hispanic White, 4.9% were non-Hispanic Black, 9.3% were Hispanic, and 16.2% were non-Hispanic Asian. Of patients without a statin prescription, the average age was 65.5 ± 14.7 years, and 58.4% were non-Hispanic White, 5.8% were non-Hispanic Black, 9.6% were Hispanic, and 13.6% were non-Hispanic Asian.
Recruitment	Not applicable
Ethics oversight	The study was approved by the Stanford University Institutional Review Board (Protocol 47644). Informed consent was waived under exemption 4: research on existing data.

Note that full information on the approval of the study protocol must also be provided in the manuscript.

Field-specific reporting

Please select the one below that is the best fit for your research. If you are not sure, read the appropriate sections before making your selection.

☒ Life sciences ☐ Behavioural & social sciences ☐ Ecological, evolutionary & environmental sciences

For a reference copy of the document with all sections, see nature.com/documents/nr-reporting-summary-flat.pdf

Life sciences study design

All studies must disclose on these points even when the disclosure is negative.

Sample size	For NLP training, validation, and application, we included the entire local population of ASCVD patients who met our study criteria of missing statin prescriptions and the presence of statin discussions in their notes. No patients who met study criteria were excluded.
Data exclusions	No data were excluded from analysis in patients who met our study criteria.
Replication	We performed bootstrap resampling of 1000 iterations to repeatedly assess NLP performance and obtain uncertainty intervals.
Randomization	Not applicable
Blinding	Not applicable

Reporting for specific materials, systems and methods

We require information from authors about some types of materials, experimental systems and methods used in many studies. Here, indicate whether each material, system or method listed is relevant to your study. If you are not sure if a list item applies to your research, read the appropriate section before selecting a response.

Materials & experimental systems

n/a	Involved in the study
☒	☐ Antibodies
☒	☐ Eukaryotic cell lines
☒	☐ Palaeontology and archaeology
☒	☐ Animals and other organisms
☒	☐ Clinical data
☒	☐ Dual use research of concern

Methods

n/a	Involved in the study
☒	☐ ChIP-seq
☒	☐ Flow cytometry
☒	☐ MRI-based neuroimaging
