# Supplementary material for: Using deep learning-based natural language processing to identify reasons for statin nonuse in patients with atherosclerotic cardiovascular disease
Source: Commun Med (Lond). 2022 Jul 15;2:88. doi: 10.1038/s43856-022-00157-w (PMC9287295; doi:10.1038/s43856-022-00157-w)
Supplement: Supplementary file 4 — Description of Additional Supplementary Files [file 43856_2022_157_MOESM4_ESM.pdf]

Description of Additional Supplementary Files

File Name: Supplementary Data 1

Description: Types of clinical notes included for analysis.
The above list outlines the titles of clinical note categories from the Stanford Healthcare Alliance (SHA) health system that were included for this study. These notes include history and physical (H&P) notes, progress notes, discharge notes, clinic notes and telephone notes.
